# Gender Differences in Psycho-Social-Spiritual Healing

**DOI:** 10.1089/jwh.2019.7837

**Published:** 2019-11-12

**Authors:** María José Luna, Rezvan Ameli, Ninet Sinaii, Julia Cheringal, Samin Panahi, Ann Berger

**Affiliations:** ^1^ Department of Psychiatry and Behavioral Sciences, Northwestern University Feinberg School of Medicine, Chicago, Illinois.; ^2^ National Institute of Mental Health, National Institutes of Health, Bethesda, Maryland.; ^3^ Clinical Center, National Institutes of Health, Bethesda, Maryland.; ^4^ Walter Reed National Military Medical Center, Bethesda, Maryland.

**Keywords:** NIH-HEALS, gender differences, connection, trust, reflection

## Abstract

***Background:*** Many individuals exhibit significant distress in response to serious and/or life-limiting illness. However, there are others who make life-transforming changes, which involve healing experiences in the psychological, social, and spiritual domains of life regardless of illness outcome. The aim of the present study is to determine if there are any differences in psycho-social-spiritual healing between genders.

***Materials and Methods:*** The NIH Healing Experiences in All Life Stressors (NIH-HEALS), a 35-item measure of psycho-social-spiritual healing, is composed of three factors: Connection, Reflection & Introspection, and Trust & Acceptance. NIH-HEALS and a demographic questionnaire were administered to 193 patients with serious and/or life-limiting illness at the National Institutes of Health Clinical Center.

***Results:*** In response to NIH-HEALS, men and women significantly differed on the Reflection & Introspection factor. Women reported increased enjoyment of mind–body practices (*p* < 0.001), compassion (*p* = 0.005), gratitude (*p* = 0.014), and a desire to be more positive (*p* = 0.044) compared to men. Men rated their pain levels (*p* = 0.035) and severity of illness (*p* = 0.016) higher and their overall level of health (*p* = 0.010) poorer compared to women. Women's responses to items regarding compassion (*r*_s_ = 0.37, *p* < 0.001) and gratitude (*r*_s_ = 0.24, *p* = 0.015) correlated positively with better overall health ratings.

***Conclusion:*** Men and women show some differences in their self-reported psycho-social-spiritual healing, which may have implications when designing interventions aimed at promoting a healing experience in the context of serious and life-limiting illness.

## Introduction

Many patients with severe and/or life-limiting illnesses can experience persistent psychosocial and spiritual distress in response to their illness.^1–4^ However, there are reports of patients who are able to make positive, life-transforming changes (LTCs), regardless of illness outcomes.^5,6^ Qualitative interviews of cancer and cardiac event survivors revealed that LTCs are characterized by changes in the psychological, social, and spiritual domains of life. For example, patients described an increase in, and strengthening of, inner resources, as well as a greater ability to function despite their illness.^7,8^ Notably, the results of LTCs expanded beyond the circumstances of the illness and positively impacted other difficult life situations such as divorce or career change.^7,8^ These LTCs are hypothesized to comprise the process of psycho-social-spiritual healing^9^ and are similar to other concepts described in the literature, such as posttraumatic growth and benefit finding.^10–12^

Gender is an important factor in psychosocial and spiritual distress and well-being in patient populations. Women with various cancer types and chronic illnesses (such as HIV) report high levels of anxiety, depression, and posttraumatic stress symptoms compared to men.^13,14^ Gender has been implicated in the experience of spiritual distress in inpatients as well.^3^ For example, women have reported higher levels of spiritual well-being than men in a study of cancer survivors.^15^ In terms of well-being, men and women identified different positive aspects of their illness in a qualitative study of over 5000 cancer patients from the American Cancer Society Study of Cancer Survivors-II (SCS-II).^16^ The positive aspects reported by women in the SCS-II include recognizing the importance of: appreciation for life, living in the present, reprioritizing what matters most in life, social support networks, developing or deepening of spirituality and existential beliefs, and finding meaning in relationships. However, the men in this study reported other positive aspects of their illness, such as feeling pleasantly surprised and grateful that their experience with cancer was better than expected and the follow-up medical surveillance of health status after the illness had been treated. Men and women thus identify distinct positive aspects of their illness and experience psychosocial and spiritual distress and well-being differently. The goal of the present study is to assess whether there are observed differences between genders in psycho-social-spiritual healing as measured by the NIH Healing Experiences in All Life Stressors (NIH-HEALS). Understanding gender differences with respect to psycho-social-spiritual healing is important in designing and implementing patient-specific interventions aimed at enhancing healing experiences.^17^

## Materials and Methods

### Participants and procedures

The study was approved by the NIH Office of Human Subject Research Protection (OHSRP). A signed written consent was waived by the OHSRP because the data were collected in an anonymous and deidentified manner. A full description of the study has been detailed elsewhere.^9^ Two hundred patients were recruited from the NIH Clinical Center inpatient and outpatient clinics. These patients were already involved in other clinical trials and research protocols for the treatment of serious, life-limiting medical illnesses. At the time of data collection, participants were in various stages of treatment and recovery. A Pain and Palliative Care Service (PPCS) representative (clinicians or Special Volunteers) approached patients in their hospital rooms or in waiting rooms of outpatient clinics. Once a patient verbally expressed interest in participating in the study, a PPCS representative verbally consented him/her to the study. The eligibility criteria for this study included age of 18 years or older, the ability to read and write in English, and the presence of a serious and/or life-limiting illness. Once consented into the study, patients completed the questionnaires on their own, while a PPCS representative remained available. Of the 200 patients that completed the questionnaire, 193 patients reported their gender. Data collected from the seven patients who did not report their gender were not included in this study. No gender identity other than man or woman was reported.

### Questionnaires

Ameli et al.,^9^ describe the battery of questionnaires administered to patients in detail. The data collected from the demographics questionnaire and NIH-HEALS were used in the present study.

The demographic questionnaire included self-report questions regarding patients' information such as age, race, ethnicity, marital status, education, religion, and employment status. In addition, it included questions regarding medical diagnosis and severity, pain severity, current and past psychiatric diagnosis and severity, stress level, social support, overall health status, and quality of life. Current and most severe medical diagnoses are listed in Table 1. They include the following: cancer, blood dyscrasias, HIV+/AIDS, genetic (such as von Hippel–Lindau Syndrome, Neurofibromatosis, Carney Complex, Job Syndrome, and X-linked severe combined immunodeficiency), and nongenetic (such as chronic Graft Versus Host Disease, Fibrous-dysplasia, Lymphangioleiomyomatosis, and autoimmune diseases) conditions.

**Table 1.** Demographics of Enrolled Participants with Serious and/or Life-Limiting Illnesses, by Gender

	*Women (*n* = 103)*	*Men (*n* = 90)*	p^a^
Age (in years)			
Mean ± SD (range)	51.0 ± 15.0 (19–89)	49.4 ± 16.2 (18–79)	0.61
Median (IQR)	52.0 (40.5–62.5)	52.5 (35.5–61.0)	
Total	96	88	
Race, *n* (%)			
American Indian/Alaska Native	0 (0)	1 (1)	0.097
Asian	9 (9)	4 (4)	
Black or African American	21 (21)	9 (10)	
Caucasian	66 (65)	73 (81)	
Mixed/two or more	3 (3)	2 (2)	
Other	3 (3)	1 (1)	
Total	102	90	
Ethnicity, *n* (%)			
Hispanic or Latinx	10 (10)	3 (3)	0.072
Not Hispanic or Latinx	90 (90)	86 (97)	
Total	100	89	
Marital status, *n* (%)			
Single	21 (21)	21 (23)	0.10
Married	62 (61)	54 (60)	
Divorced/separated	10 (10)	11 (12)	
Widowed	6 (6)	1 (1)	
Living with partner	0 (0)	3 (3)	
Other	3 (3)	0 (0)	
Total	102	90	
Religious affiliation, *n* (%)			
Christianity	68 (67)	58 (65)	0.67
Islam	1 (1)	2 (2)	
Hinduism	0 (0)	2 (2)	
Buddhism	2 (2)	0 (0)	
Judaism	2 (2)	3 (3)	
Agnostic	5 (5)	5 (6)	
Atheist	5 (5)	6 (7)	
Not affiliated	12 (12)	11 (12)	
Other	6 (6)	2 (2)	
Total	101	89	
Education, *n* (%)			
Grade school	1 (1.0)	0 (0)	0.63
High school/GED	13 (13)	12 (13)	
Vocational training	2 (2)	4 (4)	
Some college/university	21 (20)	22 (24)	
Completed college/university	33 (32)	32 (36)	
Graduate school/advanced degree	32 (31)	20 (22)	
Other	1 (1)	0 (0)	
Total	103	90	
Employment status, *n* (%)			
Full time	27 (27)	33 (37)	0.36
Part time	10 (10)	7 (8)	
Not employed	32 (31)	29 (32)	
Retired, disabled, or other	33 (32)	21 (23)	
Total	102	90	
Current, most severe medical diagnosis, *n* (%)			
HIV+/AIDS	1 (1)	1 (1)	0.97
Blood dyscrasias	9 (9)	9 (10)	
Cancer	66 (67)	63 (70)	
Genetic conditions	9 (9)	7 (8)	
Nongenetic conditions	14 (14)	10 (11)	
Total	99	90	

Percentages may not add to 100% due to rounding to the nearest whole number.

^a^ *p*-Values are from comparisons of data between genders.

IQR, interquartile range (25th to 75th percentiles); SD, standard deviation.

The NIH-HEALS is a 35-item, self-report questionnaire that measures psycho-social-spiritual healing in patients with severe and/or life-limiting illnesses. Item responses are scored on a five-point Likert scale from Strongly Disagree (1) to Strongly Agree (5). Four items require reverse scoring. This questionnaire has strong internal consistency (Cronbach's α = 0.89) and stable factor structure, and its convergent and divergent validity have been confirmed.^9^ Factor analysis of the NIH-HEALS supported a three-factor structure, that is, Connection, Reflection & Introspection, and Trust & Acceptance.

### Statistics

Data were analyzed using SAS v9.4 (SAS Institute, Inc., Cary, NC) and are reported as mean ± standard deviation or frequencies and percentages. All data were assessed for normality of distributions, and nonparametric tests were used where applicable. As applicable, two-sided *t*-tests or Wilcoxon Rank-Sum tests were used to compare continuous data (*i.e*., NIH-HEALS total scores and the scores for the Connection, Reflection & Introspection, and Trust & Acceptance factors) between genders. Fisher's exact tests compared categorical data, and the Kruskal–Wallis and Jonckheere–Terpstra tests compared singly- or doubly-ordered categorical data, respectively (*i.e*., responses between men and women to certain self-report demographics questions). Spearman's rho was used to test the correlations between NIH-HEALS items and responses to demographic questions. A two-sided *p*-value of <0.05 and a confidence interval excluding the null were considered statistically significant.

## Results

The demographic information of the subjects in this sample has been described elsewhere in detail.^9^ Ages ranged from 18 to 89 with a mean of 50.2 years (±15.5). Of those who reported their gender (*n* = 193), 47% of the sample identified as men and 53% identified as women. No other gender identities were reported. Patients were Caucasian (72%), Black or African American (16%), or Asian (7%). Of the whole sample, 7% identified their ethnicity as Hispanic/Latinx. Subjects reported their religious affiliations as Christian (66%), Not Affiliated (12%), or Atheist (6%). Of subjects who reported their educational attainment, 61% completed college/university or graduate school/advanced degree. At the time of data collection, 31% were employed full-time, 9% were employed part-time, 32% were not employed, and 28% of patients were not working due to retirement, disability, or other reasons. Table 1 summarizes the demographic information by gender. No statistically significant differences between genders were observed with regards to race, ethnicity, religious affiliation, education, employment status, and medical diagnosis.

### Gender differences in response to NIH-HEALS

Table 2 summarizes the descriptive statistics of NIH-HEALS total and individual factor scores. Men and women had similar NIH-HEALS total scores and individual factor scores for Connection and Trust & Acceptance. However, women had significantly higher scores than men on the Reflection & Introspection factor (56.1 ± 6.3 vs. 53.3 ± 7.2; *p* = 0.005). In examining individual items, men and women responded differently to four items within the Reflection & Introspection factor. In each case, women tended to endorse these four items significantly more than men. The four items were: “I enjoy activities that involve both mind/body such as meditation, prayer, yoga, tai chi, chanting” (*p* < 0.001); “Difficult circumstances in my life have increased my compassion towards others” (*p* = 0.005); “I have an increased sense of gratitude” (*p* = 0.014); and “Life challenges raised my desire to be more positive” (*p* = 0.044) (Table 3).

**Table 2.** NIH Healing Experiences in All Life Stressors Total and Factor Scores, by Gender, in Patients with Serious and/or Life-Limiting Illnesses

	n	*Mean* ± *SD*	*95% CI*	p^a^
NIH-HEALS total				
Men	85	131.0 ± 19.7	126.7–135.2	0.19
Women	97	134.6 ± 17.8	131.0–138.2	
Factor 1: Connection				
Men	89	36.9 ± 9.8	34.8–38.9	0.21
Women	101	38.3 ± 10.0	36.3–40.3	
Factor 2: Reflection & Introspection				
Men	87	53.3 ± 7.2	51.8–54.9	0.005^b^
Women	101	56.1 ± 6.3	54.9–57.4	
Factor 3: Trust & Acceptance				
Men	89	40.3 ± 7.3	38.7–41.8	0.75
Women	100	40.6 ± 6.4	39.3–41.9	

^a^ *p*-Values are from comparisons of data between genders.

^b^ Statistical significance at *p*-value <0.01.

CI, confidence interval; NIH-HEALS, NIH Healing Experiences in All Life Stressors.

**Table 3.** Gender Differences in Response to Four Specific NIH Healing Experiences in All Life Stressors Reflection & Introspection (Factor 2) Items in Patients with Serious and/or Life-Limiting Illness

*Reflection & Introspection items*							
*Items*		*Strongly disagree*	*Disagree*	*Neither agree nor disagree*	*Agree*	*Strongly agree*	p
I enjoy activities that involve both mind/body such as meditation, prayer, yoga, tai chi, chanting.	Men (*n* = 90)	9 (10)	7 (8)	31 (34)	29 (32)	14 (16)	<0.001^b^
	Women (*n* = 103)	0 (0)	6 (6)	24 (23)	41 (40)	32 (31)	
Difficult circumstances in my life have increased my compassion toward others.	Men (*n* = 89)	1 (1)	4 (4)	17 (19)	46 (52)	21 (24)	0.005^c^
	Women (*n* = 101)	0 (0)	3 (3)	9 (9)	49 (49)	40 (40)	
I have an increased sense of gratitude.	Men (*n* = 90)	0 (0)	4 (4)	21 (23)	39 (43)	26 (29)	0.014^d^
	Women (*n* = 103)	0 (0)	1 (1)	12 (12)	49 (48)	41 (40)	
Life challenges raised my desire to be more positive.	Men (*n* = 90)	0 (0)	5 (6)	24 (27)	46 (51)	15 (17)	0.044^d^
	Women (*n* = 103)	0 (0)	4 (4)	18 (18)	54 (52)	27 (26)	

Data are presented as frequencies (percentages). Percentages may not add to 100% due to rounding to the nearest whole number.

^a^ *p*-Values are from comparisons of data between genders.

Statistical significance at: ^b^*p* < 0.001; ^c^*p* < 0.01; ^d^*p* < 0.05.

### Gender differences in response to demographics questionnaire

There were no statistically significant differences between men and women in their responses to questions regarding history of, or current, psychiatric illness, current psychiatric illness severity, current level of stress, current level of social support, and quality of life (Table 4). However, men tended to rate the severity of their medical illness (*p* = 0.016) and current pain levels (*p* = 0.035) as worse than women. Women tended to rate their current overall health status (*p* = 0.010) as better than did men (Table 4).

**Table 4.** Responses to Select Items from the Demographics Questionnaire in Men and Women with Serious and/or Life-Limiting Illness

						p^a^
Do you have a history of psychiatric illness?						
	Yes	No				0.26
Men (*n* = 89)	13 (15)	76 (85)				
Women (*n* = 101)	9 (9)	92 (91)				
Do you have a current psychiatric diagnosis?						
	Yes	No				0.45
Men (*n* = 90)	17 (19)	73 (81)				
Women (*n* = 102)	15 (15)	87 (85)				
If yes, indicate severity of your current psychiatric illness:						
	Not severe	Mild	Moderate	Severe		0.18
Men (*n* = 16)	3 (19)	3 (19)	8 (50)	2 (13)		
Women (*n* = 14)	1 (7)	10 (71)	2 (14)	1 (7)		
What is your current level of social support (friends, family, community, religion/spirituality, other)?						
	No support	Some support	Good support	Excellent support		
Men (*n* = 85)	0 (0)	15 (18)	24 (28)	46 (54)		0.30
Women (*n* = 102)	0 (0)	11 (11)	30 (29)	61 (60)		
Please rate your current level of stress:						
	No stress	Mild	Moderate	Severe	Extreme	
Men (*n* = 89)	9 (10)	27 (30)	39 (44)	10 (11)	4 (4)	0.91
Women (*n* = 97)	9 (9)	28 (29)	49 (51)	10 (10)	1 (1)	
How severe is your medical illness?						
	Not severe	Mild	Moderate	Severe	Extremely severe/life-limiting	
Men (*n* = 89)	1 (1)	2 (2)	12 (13)	28 (31)	46 (52)	0.016^b^
Women (n = 98)	2 (2)	8 (8)	15 (15)	39 (40)	34 (35)	
Are you experiencing pain?						
	No pain	Some pain	Moderate pain	Severe pain	Extremely severe pain	
Men (*n* = 90)	23 (26)	17 (19)	22 (24)	23 (26)	5 (6)	0.035^b^
Women (*n* = 101)	31 (31)	32 (32)	20 (20)	14 (14)	4 (4)	
How do you rate your current overall health status?						
	Poor	Manageable	Satisfactory/fair	Good	Excellent	
Men (*n* = 87)	15 (17)	43 (49)	13 (15)	14 (16)	2 (2)	0.010^c^
Women (*n* = 102)	11 (11)	34 (33)	32 (31)	23 (23)	2 (2)	
How do you rate your quality of life?						
	Poor	Manageable	Satisfactory/fair	Good	Excellent	
Men (*n* = 87)	7 (8)	22 (25)	22 (25)	23 (26)	13 (15)	0.055
Women (*n* = 101)	8 (8)	14 (14)	18 (18)	46 (46)	15 (15)	

Data are presented as frequencies (percentages). Percentages may not add to 100% due to rounding to the nearest whole number.

^a^ *p*-Values are from comparisons of data between genders.

Statistical significance at: ^b^*p* < 0.05; ^c^*p* < 0.01.

### Relationship between NIH-HEALS items and self-reported severity of illness, severity of pain, and overall health status

The NIH-HEALS Reflection & Introspection items for which there were statistically significant differences between men's and women's responses were assessed in relation to questions regarding overall health, severity of illness, and current pain level ratings. There was a tendency for women to agree with two NIH-HEALS items, namely “Difficult circumstances in my life have increased my compassion towards others” (*r*_s_ = 0.37, *p* < 0.001) and “I have an increased sense of gratitude” (*r*_s_ = 0.24, *p* = 0.015), each with reports of better overall health status.

## Discussion

This study evaluated the differences between men and women with severe and/or life-limiting illnesses in response to NIH-HEALS, a measure of psycho-social-spiritual healing. Although there were no significant differences between genders in the NIH-HEALS total score, women scored significantly higher on the Reflection & Introspection factor. No differences were found in response to the Connection and Trust & Acceptance factors. Women agreed with Reflection & Introspection items regarding a sense of compassion toward others, gratitude, enjoyment of activities that involve the mind and body, and a desire to be more positive. Items related to compassion and gratitude positively correlated with self-reported overall health status in women.

The Connection factor of NIH-HEALS includes items that have to do with a sense of connection to community, family, and/or a higher power. We did not observe differences between men and women in our sample in response to the Connection factor. This is consistent with previous studies that identified connection with a higher power, religion, and or significant others as important resources that enhance coping and quality of life in both genders. Social support and spiritual or religious coping—the processes through which patients harness their beliefs and traditions as psychological resources—are commonly reported as key resources that are helpful in coping with stressful life events, such as serious life-limiting illness.^18–20^ Spiritual and religious coping are predictors of mental health for men and women with significant health conditions such as cancer, traumatic brain injuries, and stroke.^21^ Support from cancer patients' religious communities is associated with increased quality of life for both genders.^22^ Similarly, evidence suggests that social support for breast and prostate cancer patients is a significant variable in patients' distress.^23,24^ In patient samples that include men and women, social support, particularly that which is found within the connection with family, is a predictor of well-being.^25,26^

The Trust & Acceptance factor is composed of items that capture acceptance of the current situation and trust that support networks and medical caregivers will be responsive to one's needs. We did not find gender differences in response to the Trust & Acceptance factor. Other studies identified trust and acceptance as important factors involved in patients' well-being. Helgeson et al.^11^ examined the relationship between positive changes made in response to a traumatic event (including serious illness) and psychological and health outcomes. In this study, acceptance was found to be a correlate of physical and psychological well-being for men and women. In addition, patients from the SCS-II study identified Medical Support as an important theme of positive aspects of having cancer. Both men and women endorsed Medical Support items that describe support and reliable relationships with medical care providers.^16^

The difference between genders in the Reflection & Introspection factor of NIH-HEALS in the present study was due to differences in response to four items that capture compassion, gratitude, enjoyment of activities that involve the mind and body, and a desire to be more positive. Patients of both genders have conveyed the importance of compassion when coping with their illness.^16,27,28^ The present study found that women expressed more compassion in response to NIH-HEALS, suggesting that perhaps women in our sample may more readily identify compassion for others in their healing process than men. However, this does not mean that this construct is unimportant in men. Psychometric testing of a revised version of the Posttraumatic Growth Inventory (PTGI)^29^ with a sample of prostate cancer survivors revealed that five newly added items regarding compassion was loaded onto a novel sixth factor, named Compassion, which accounted for about 50% of the overall variance in response to the PTGI.^30^ This indicates that compassion is important to men's well-being during and after cancer. Therefore, compassion may be influential to the process of psycho-social-spiritual healing in both men and women, although possibly to different degrees.

While some studies found gratitude among genders to be similar,^31^ others have found that women are more likely to feel, express, and derive psychological benefits from gratitude compared to men.^32^ Studies of breast cancer patients and survivors indicate that increased gratitude during and after recovery from illness was positively associated with perceived social support, well-being, and posttraumatic growth and negatively associated with distress.^33,34^ Similar to the concept of compassion, the presence of gratitude in women and/or their ability to express it does not preclude its importance for men. Indeed, in a study of heart failure patients, in which 95% were men, Mills et al. (2015) found that gratitude was positively associated with better sleep, mood, self-efficacy, and lower inflammation.^35^ Moreover, authors report that increased gratitude mediated patients' spiritual well-being, which also impacted sleep and mood positively. The SCS-II study found that both men and women identify increased gratitude as a positive aspect of having cancer; however, the context in which gratitude had a positive effect differed between genders.^16^ Women were more likely to endorse gratitude in respect to “appreciating, valuing, and enjoying life,” while men endorsed gratitude specifically to “the diagnostic and treatment phase of cancer.” Previous research indicates that while expression of gratitude may be lower in men, gratitude does play a role in their health and well-being. It is therefore important to consider patients' gender and context when considering interventions focused on gratitude to maximize psycho-social-spiritual healing experiences.

Women's endorsement of NIH-HEALS compassion and gratitude items correlated positively with their self-reported overall health. This observation is consistent with previous research linking compassion and gratitude with health and well-being.^36–38^ Indeed, compassion and gratitude have been recognized as influential factors involved in constructs related to psycho-social-spiritual healing, such as posttraumatic growth.^37^ PTG has been demonstrated to correlate positively with health and well-being and negatively with posttraumatic stress symptoms in patients with serious illnesses.^39^ For example, cancer survivors identified the newfound compassion for others as a result of their own diagnosis to be of particular salience.^37^ Similarly, gratitude has been identified as a psychological correlate of PTG^39^ and emerged as an important theme of positive aspects of having had cancer.^16^

The finding that women tended to agree more strongly than men with the NIH-HEALS item “I enjoy activities that involve both the mind and body such as meditation, prayer, yoga, tai chi, chanting” is consistent with previous research. It has been reported that women with cancer pray for their health more than men.^40^ Men are less likely to use complementary, alternative, and integrative medicine approaches, including meditation^41^ and yoga.^42^

Women in our sample also tended to endorse the item “Life challenges raised my desire to be more positive” more highly than men. Optimism and positive reappraisal in the context of illness have been found to significantly correlate with posttraumatic growth and related construct “benefit-finding” in both genders,^11,43^ as well as in women survivors of breast cancer.^39^ Again, both men and women may benefit from interventions that enhance a desire to be positive despite their illness trajectory. It is also possible that these interventions may need to be tailored with sensitivity to gender differences and the ease with which each gender may incorporate and respond to them.

We also observed differences between men and women in response to self-report health-related questions. Men rated their severity of illness higher than women, which is congruent with previous research demonstrating that women perceive their illness to be less severe than men.^44,45^ Women in this study also reported less pain than men. While this is consistent with reports that gender can affect pain experiences, there are reports that have demonstrated that men experienced less pain more often than women.^46–48^ In our sample, psychiatric diagnoses, psychiatric diagnosis severity, social support, and quality of life did not differ between genders. However, there are studies that have demonstrated associations between gender and psychiatric comorbidities, perceived social support, and quality of life.^49–53^

There are several limitations to this study. NIH-HEALS was developed and validated in a clinical research setting in the United States.^9^ The observed differences in psycho-social-spiritual healing between genders may not be generalizable internationally due to the variation in the conceptualization in healing across cultures.^54^ Similarly, at the national level, these results should be interpreted with caution. It should be emphasized that the NIH Clinical and Research Center is a unique setting where patients with severe and rare diseases are treated in ways that may not resemble other academic or community medical centers.^55^ For example, unlike most hospitals, patients are enrolled in protocols that allow inpatient, day hospital, and outpatient visits in a relatively fluid manner, and therefore, the distinction between inpatient and outpatient enrollment may not reflect meaningful differences regarding the nature of treatments or severity of illness as in other community or hospital settings. Another limitation is the subjects' educational level. More than half of our sample has high levels of educational attainment. In contrast, about one third of the general population in the United States have received at least a bachelor's degree.^56^ The results of this study are intended to eventually guide the development and implementation of gender-sensitive healing interventions. Because normative data for NIH-HEALS in clinical and subclinical populations are not yet available, use of NIH-HEALS for individual assessments in clinical practice is not yet recommended.^9^

Patients in our sample did not report any nonbinary gender identities. Given the health disparities, discrimination, and stigma that gender minorities experience in the health care system and the subsequent mistrust of medical professionals that arises in the community,^57,58^ it is possible that psycho-social-spiritual healing may be influenced by other nonbinary gender identities. Future studies should investigate the processes involved in psycho-social-spiritual healing in individuals who identify with nonbinary gender identities. In addition, we did not explore the ways in which gender roles and expectations intersect with gender identity to influence healing experiences and self-reported pain, severity of medical illness, and perception of overall health. Future studies are needed to elucidate the mechanism(s) underlying the gender differences in the process of psycho-social-spiritual healing to maximize and optimize interventions to enhance well-being and reduce distress for patients with serious, life-limiting illnesses.

## Conclusion

While men and women in our study have endorsed an overall similar level of psycho-social-spiritual healing, they may experience reflective and introspective processes of healing differently. This difference is particularly salient in the domains of compassion, gratitude, participating in activities that involve the mind and body, and desire to be positive. Despite these differences, evidence from the literature supports that compassion, gratitude, and desire to be positive are relevant to both men and women. Patients of both genders exhibiting psycho-social-spiritual distress may therefore benefit from interventions that promote these qualities, which, in turn, may improve their perceptions of their overall health. In addition, data on health benefits of alternative and integrative approaches such as yoga, meditation, and mindfulness approaches to health and well-being are mounting.^59–62^ It is, therefore, important to better understand the reasons for gender disparity in valuing, accepting, and utilizing these approaches and possibly recognize that in clinical settings men may require more education from their health care providers regarding the benefits of these approaches.
